# Polycationic doping of the LATP ceramic electrolyte for Li-ion batteries†

**DOI:** 10.1039/d2ra05782d

**Published:** 2022-10-17

**Authors:** Aiym Mashekova, Yelnury Baltash, Mukagali Yegamkulov, Ivan Trussov, Zhumabay Bakenov, Aliya Mukanova

**Affiliations:** Institute of Batteries LLC Kabanbay Batyr Ave. 53 Nur-Sultan 010000 Kazakhstan zbakenov@nu.edu.kz aliya.mukanova@nu.edu.kz; Department of Chemical and Materials Engineering, School of Engineering and Digital Sciences, Nazarbayev University Kabanbay Batyr Ave. 53 Nur-Sultan 010000 Kazakhstan; National Laboratory Astana, Nazarbayev University Kabanbay Batyr Ave. 53 Nur-Sultan 010000 Kazakhstan

## Abstract

All-solid-state Li-ion batteries (LIBs) with a solid electrolyte instead of a liquid one demonstrate significantly higher safety in contrast with the conventional liquid-based LIBs. An inorganic NASICON-type Li conductor Li_1.3_Al_0.3_Ti_1.7_(PO_4_)_3_ (LATP) is a promising solid electrolyte with an ionic conductivity of up to 10^−3^ S cm^−1^ at room temperature. However, LATP gradually degrades in contact with Li metal because of reduction of Ti^4+^ to Ti^3+^, resulting in a lower ionic conductivity at the electrolyte–electrode interface. Cation doping is a promising approach to stabilize the LATP structure and mitigate the Ti reduction. Here, we report our findings on the alternative polycationic doping strategy of the LiTi_2_(PO_4_)_3_ (LTP) structure, when a heterovalent cation is added along with Al. In particular, we studied the effect of tetravalent and divalent cation dopants (Zr, Hf, Ca, Mg, Sr) of LATP on the Li-ion conduction and Ti reduction during interaction with lithium metal. The samples were prepared by molten flux and solid-state reaction methods. The structure, morphology, and ion-transport properties of the samples were analyzed. The activation energy of Li-ion migration in all synthesized systems was calculated based on the electrochemical impedance spectroscopy (EIS) data retrieved for a temperature range of 25–100 °C. From the obtained results, the tetravalent doping (Zr^4+^ and Hf^4+^) appeared to be a more promissing route to improve the LATP electrolyte than the divalent doping (Mg^2+^, Ca^2+^, and Sr^2+^). The X-ray photoelectron spectroscopy analysis of the samples after their contact with lithium provided the data, which could shed light on the effect of the incorporated dopants onto the Ti reduction.

## Abstract 2

The tetravalent cations with similar sizes (Zr^4+^, Hf^4+^) supress the Ti^4+^ reduction while the bulky divalent cations (Ca^2+^, Sr^2+^) promote it even at relatively low concentrations.

## Graphical abstract

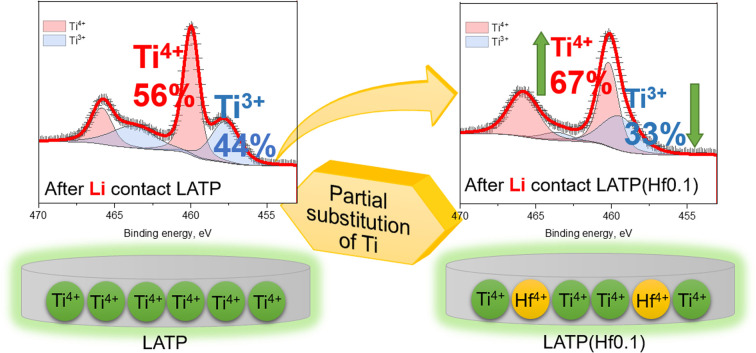

## Introduction

Lithium-ion batteries (LIBs) are characterized by high specific energy, high efficiency and long service life. These properties have helped LIBs to become the most preferred power source for the consumer electronics market with a production volume of about a billion cells per year.

Existing commercial LIBs containing liquid electrolytes do not fully ensure the safety of an electronic device due to a number of disadvantages of liquid electrolytes, including the safety concerns due to possible leakage, flammability, electrochemical instability, *etc.*^1,2^ The use of a solid-phase electrolyte not only improves safety of LIBs, but also increases their service life by suppressing degradation processes. Solid-state inorganic batteries show significant advantages in terms of electrochemical stability window, operating temperature range and safety over conventional liquid electrolyte systems.^3–5^ Completely inorganic solid-state batteries based on inorganic amorphous or ceramic solid electrolytes (SE) contain no flammable components and have a high stability.^6^

SE with NASICON-type structure not only possess the lowest activation energy for the Li-ion conduction but also exhibit stability towards air and water, and has a high Li-ion room temperature conductivity in the range of 10^−6^–10^−3^ S cm^−1^.^7–10^ The NASICON-type electrolytes with the general formula of NaM_2_(PO_4_)_3_ usually crystallize in a trigonal crystal symmetry. The structure is distinguished by the presence of a system of cavities, forming a three-dimensional network of channels through which the movement of alkali ions is possible. Small changes in this structure, such as heterovalent doping, can lead to a change in SE properties. Among a wide variety of SE NASICONs, LiTi_2_(PO_4_)_3_ (LTP) was marked as a material exhibiting high chemical and thermal stability with a total ionic conductivity of 10^−6^ S cm^−1^. A heterovalent substitution with Al cation (0.3 Al per formula unit) into the Ti site increased the conductivity up to 10^−3^ S cm^−1^.^11–16^ The resulting compound remained the NASICON structural type and was described by a general formula Li_1+*x*_Al_*x*_Ti_2−*x*_(PO_4_)_3_ (LATP).^17^ However, in contact with Li metal LATP's surface gradually degrades due to Ti^4+^ reduction to Ti^3+^, resulting in a lowered ionic conductivity at the electrolyte–electrode interface that delays or even stops the migration of Li-ions. Compounds like LiM_2_(PO_4_)_3_ (M = Zr and Hf) are isostructural to LATP, while Hf and Zr are being unaffected by metallic Li reduction.^18–20^ Compared to Ti^4+^ (ionic radius 0.605 Å), the ionic radii of Zr^4+^ and Hf^4+^ are larger (0.72 Å and 0.71 Å respectively). It was shown that the variation of average M^4+^ cation ionic radius affects the electrochemical properties of LiM_2_(PO_4_)_3_ (M = Hf or Zr, Ge).^21^ Additionally, alkaline earth materials are one type of widely used doping elements because of their abundant reserves compared with rare earth material. For example, doping Li_1.5_Al_0.5_Ge_1.5_(PO_4_)_3_ with Mg leads to an increase in the concentration of Li-ions and the expansion of Li-ion conducting channels because the ionic radius of Mg^2+^ (0.72 Å) is larger than that of Ge^4+^ (0.53 Å) and Al^3+^ (0.54 Å). The ionic conductivity of Li_1.6_Al_0.4_Mg_0.1_Ge_1.5_(PO_4_)_3_ was shown to be 7.4 × 10^−3^ S cm^−1^ and is greater than the ionic conductivity of Li_1.5_Al_0.5_Ge_1.5_(PO_4_)_3_–2.9 × 10^−3^ S cm^−1^.^22^ Similarly, Al^3+^ in the LATP can be partially replaced by Mg^2+^. The reduction of the total positive charge requires the insertion of additional Li-ions into the crystal lattice to maintain electroneutrality. This, in turn, supposedly can have a positive effect on conductivity due to an increase in the number of charge carriers. Similar substitution strategies were carried out with other divalent metals with similar properties such as Ca^2+^, Sr^2+^.^23–25^ Presumably, the larger size of Sr^2+^ compared to Al^3+^ allows increasing the crystal lattice and thereby facilitating the migration of Li-ions. At the same time, the Sr^2+^ cation (smaller than Al^3+^) allows, based on the condition of electroneutrality, to obtain compounds with an increased concentration of Li-ion carriers. As a result, with the partial replacement of Al^3+^ by Sr^2+^ in LATP, an increase in the ionic conductivity can be expected.

However, although the study of NASICON doping *via* different strategies is a quite mature subject, the investigation of simultaneous presence of doping agents and Al in LATP remains rather incomplete. Therefore, it is of interest to investigate the possibility of heterovalent partial cationic substitution in Al-containing NASICONs.

In this work, we focus on the synthesis of LATP with partial substitution of Ti and/or Al with tetra-(Zr and Hf) and di-(Mg, Sr, Ca) valent cations to investigate their effect on the electrolyte performance. Due to the synthesis complexity specific to choice of the precursors two most commonly used synthesis methods were chosen: molten flux (where molten flux as the reaction medium can maintain the desired liquid medium to increase the ion transfer rate and decrease the temperature and reaction time) and solid-state reaction method (where the precursors are milled).^26^ Moreover, this research presents the comprehensive study of the effect of doping on the ionic conductivity of modified LATP as well on the suppression of Ti^4+^ reduction at the contact with Li metal.

## Experimental

### Material synthesis

The samples with a tetravalent cation substitution were synthesized according to a general formula Li_1.3+*х*_Al_0.3_M_*x*_Ti_1.7−*x*_(PO_4_)_3_, where M = Zr, Hf. The materials with a divalent cation substitution were synthesized according to a general formula Li_1.3+*х*_Al_0.3−*x*_M_*x*_Ti_1.7_(PO_4_)_3_, where M = Mg, Ca, Sr. The samples are further indicated as LATP(M_*x*_), where M is doping cation and *x* is its amount. The samples were prepared *via* molten flux method by Liu *et al.*^13^ LiNO_3_ (97%), Al_2_O_3_ (99%), TiO_2_ (99.7%), and a doping agent from ZrO_2_ (99%), HfO_2_ (98%), MgO (99.9%), Ca(OH)_2_ (96%), or SrO (99.9%), and NH_4_H_2_PO_4_ (99%) were used as precursors provided by Sigma Aldrich. Additional 10% of Li excess was added to all samples at the initial stage of preparation of the precursors to mitigate the loss of Li during sintering.^27–29^ Firstly, water-soluble precursors (LiNO_3_, NH_4_H_2_PO_4_) were rigorously stirred in deionized water until full dissolution. After that metal oxides and urea (NH_2_)_2_CO (99.5%) (doubled amount of the loaded NH_4_H_2_PO_4_) were added to the solution. The total mass of the dry components was about 20 g, and the water volume was 25 mL. The prepared solution was mixed using a magnetic stirrer at 70 °C for 12 h until dry. Then the resultant mixture was annealed in air at 600 °C for 8 h. The obtained product was ground in an agate mortar, and the resulting crystalline powder was pressed into 10 mm diameters pellets with pressure of 10 tons (1.25 GPa). Further, the pellets were sintered at 800 °C for 6 h in air. Also, due to the difficulties during preparation, the samples with the divalent cation substitution were prepared *via* conventional solid-state route with the same reagents with intermediate treatment at 600 °C for 8 h and a subsequent sintering at 800 °C for 6 h in air.

### Characterization

The obtained samples were analysed by X-ray diffraction (XRD) on Rigaku Smart Lab diffractometer with a copper X-ray source (*λ*_CuKα1_ = 1.54056 Å, *λ*_CuKα2_ = 1.54439 Å) in a Bragg–Brentano reflection geometry equipped with D/teX Ultra 250 detector. Structural refinement was performed by the Rietveld method with the GSASII software.^30^ The ionic conductivity of pelletized samples was measured by an electrochemical impedance spectroscopy (EIS) using Metrohm AutoLab 204 in a frequency range of 1 Hz to 1 MHz with 10 mV potential amplitude. Ionic conductivity of the electrolyte pellets was calculated using the equation *σ* = *d*/*RS*, where *σ* is the ion conductivity, *d* is a thickness, *R* is a resistance and *S* is the area of a pellet. The obtained data were normalized to a pellet with an area of 1 cm^2^ and a thickness of 0.1 cm. Gold as a blocking electrode was deposited onto both sides of the pellets by magnetron sputtering (Angstrom Engineering). The sputtering was conducted at 5 mTorr for 15 min and the thickness of coated Au was around 100 nm. The ionic conductivity measurements were repeated at different temperatures (25, 50, 60, 80 and 100 °C) to determine the activation energy of ion migration in the electrolyte using the Arrhenius equation for conductivity.^31^ In order to ensure thermal equilibration, the samples were kept for 30 min at each temperature for stabilization before the measurements. Morphology and grain size of the electrolytes were investigated on Zeiss Crossbeam 540 scanning electron microscope (SEM). Elemental analysis of doped LATP was carried out using energy dispersive X-ray spectroscopy (EDS). In order to test the stability against lithium metal, the obtained electrolytes were placed between two Li-metal chips in split cells in Ar-filled glow-box (MBraun LabMaster). Then the EIS measurements were taken and the electrolytes were left in a split cell for 20 hours. After this the surface of doped LATP was investigated by X-ray photoelectron spectroscopy (XPS) on a spectrometer the NEXSA XPS, which was equipped with an Al_Kα_ achromatic X-ray source (1486.6 eV).

## Results and discussion

### Structural characterisation

**Fig. 1:**
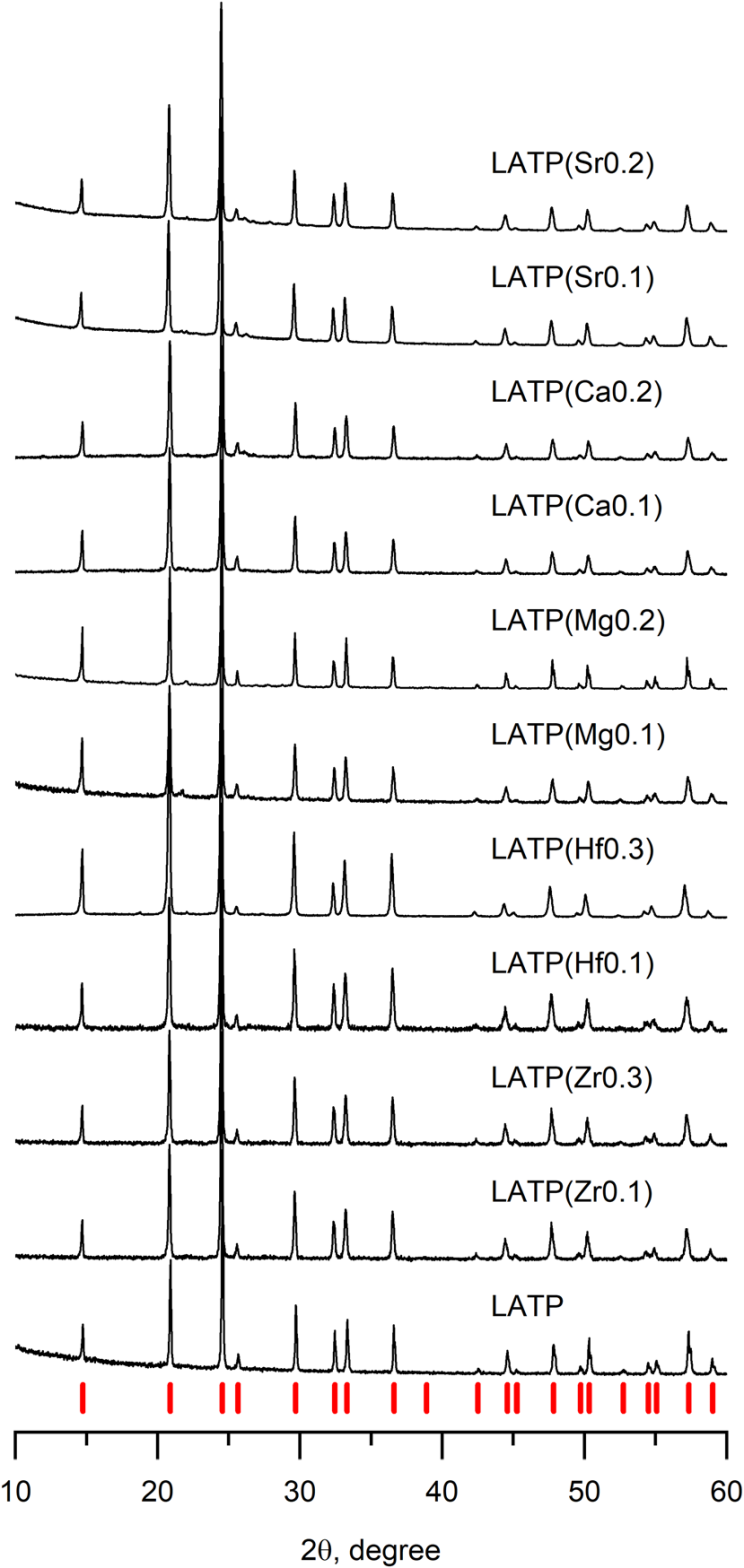
Diffraction patterns of doped LATP samples with various levels of cation substitutions. Values in brackets designate the type and amount of doping ion. Red tick-marks represent NASICON phase with space group *R*3̄*c*.

In order to identify whether the obtained samples have the NASICON structure, the XRD analysis was performed; collected data are presented in Fig. 1. Among several selected concentrations of tetravalent and divalent cation dopants, pure NASICON-type structure was obtained only in the cases where the atomic fraction of the dopants was no more than *x* = 0.3, where *x* is atomic unit of a doping cation (examples of XRD of samples with higher *x* are provided in the ESI, Fig. S1†). The diffraction plots of all synthesized samples in Fig. 1 demonstrate a typical pattern corresponding to a NASICON structure with a trigonal space group *R*3̄*c* (the corresponding peaks are indicated by red tick-marks at the bottom). The XRD patterns with tetravalent cation doping (Zr^4+^, Hf^4+^) show no impurities suggesting successful synthesis of the desired compositions. It was reported earlier that purely Zr-based LTP crystallizes into a monoclinic crystal system instead of trigonal resulting in the peak splitting on the XRD pattern.^18–20^ However, at the presented levels of doping, there was no any splitting peak suggesting no change of symmetry after the doping. Besides, introduction of higher concentrations of dopants (*x* > 0.3) led to a noticeable phase separation. One of the main obtained side-products were LiZr(PO_4_) (*Pbna*), LiZr_2_(PO_4_)_3_ (*P*21/*n*) and AlPO_4_. A phase separation is somewhat surprising for these compositions since Al-free phases are known to the date. The key to this phenomenon could be an instability introduced by a smaller Al^3+^ cation in the presence of bulky Zr^4+^ or Hf^4+^. Similar systems of Ga–Zr demonstrate more stability, apparently due to a larger Ga ion (*r*_Ga^3+^_ = 0.62 Å).^32,33^

The study of the divalent cation doping of LATP was performed in a similar manner as above. It was experimentally found that the molten flux method is unsuitable for obtaining the pure NASICON phase of Ca^2+^, Mg^2+^, and Sr^2+^ doped systems. Supposedly, it is because urea forms relatively strong complexes with alkali earth metals, therefore, inducing their separation from the rest of the reagents thus causing the observed formation of some the TiP_2_O_7_ impurity phase. Therefore, in order to avoid such complex formation, the synthesis method was switched to the solid-state route.

The diffractograms of the samples (Fig. 1) synthesized by solid-state reaction (divalent cation doping) show the formation of a pure LATP phase at *x* = 0.2 doping level without any impurities. However, when the amount of doping cation was above *x* = 0.2, an additional phase of LiTiPO_5_ appears suggesting close solubility limits of the alkali earth cation content or thermodynamic instability of this phase at higher temperatures (Fig. S2†).

In order to check the distribution of the elements in the obtained samples and to look for potential impurities, the SEM-combined EDX was performed (Fig. S4 and S5†). It was observed that the samples had an even elemental distribution, indicating the successfully completed doping process. Moreover, it demonstrated that there are no phase impurities in the samples that could be inaccessible by the XRD technique.

To further study the samples and investigate if doping affects the crystal structure, the lattice parameters were refined by the Rietveld method. The refined cell parameters of undoped LATP were used as a reference point (Table 1). The cell parameters expectedly increase with the increase of the amount of doping cation for both Zr and Hf-based systems. Taking into account that the incorporation of larger atoms normally causes the expansion of the unit cell and the atomic radius for Ti (*r*_Ti^4+^_ = 0.605 Å) is smaller than that of Zr or Hf (*r*_Zr^4+^_ = 0.72 Å, *r*_Hf^4+^_ = 0.71 Å), the observed cell expansion suggests a successful incorporation of dopants into the structure. The cell parameters of both Zr and Hf containing samples with the same levels of doping are very similar due to the same sizes for Zr^4+^ and Hf^4+^.^21^ A similar observation was made for Al-free LTP systems doped with Zr^4+^ and Hf^4+^.^18–20^

**Table 1:** Refined lattice parameters of the obtained samples

Sample	*a*, Å	*c*, Å	Volume, Å^3^
LATP	8.5069(1)	20.8679(4)	1307.83(9)
LATP(Zr_0.1_)	8.5196(4)	20.9154(3)	1314.74(1)
LATP(Zr_0.3_)	8.5454(1)	20.9972(4)	1327.87(6)
LATP(Hf_0.1_)	8.5230(2)	20.9387(9)	1317.25(4)
LATP(Hf_0.3_)	8.5412(5)	20.9862(7)	1325.89(4)
LATP(Mg_0.1_)	8.5087(3)	20.8381(6)	1307.51(7)
LATP(Mg_0.2_)	8.5119(1)	20.8554(5)	1308.58(6)
LATP(Ca_0.1_)	8.5063(5)	20.9265(5)	1311.31(9)
LATP(Ca_0.2_)	8.5294(6)	20.9824(1)	1321.97(1)
LATP(Sr_0.1_)	8.5103(6)	20.8644(2)	1308.65(8)
LATP(Sr_0.2_)	8.5156(1)	20.8679(4)	1310.50(9)

According to the results of the structural Rietveld refinement, a rather insignificant change in the cell parameters was observed for divalent cation doping (Table 1). This observation is unexpected since a partial substitution of Al ions with the atomic radius (*r*_Al^3+^_ = 0.535 Å) in the system Li_1.3+*х*_Al_0.3−*x*_M_*x*_Ti_1.7_(PO_4_)_3_, where *x* = 0.1 and 0.2 by Ca, Mg and Sr cations (*r*_Ca^2+^_ = 0.99 Å, *r*_Mg^2+^_ = 0.72 Å, *r*_Sr^2+^_ = 1.12 Å) should cause more significant unit cell expansion. Taking into account that the doping was done with a step of 0.1 a.u., 33% relative to Al content and 5% relative to Ti site are substituted. If there is no doping, such significant change would result in a noticeable number of impurities in the XRD pattern. Additionally, the EDS mapping (Fig. S3 and S4†) does not show the presence of amorphous impurities, which cannot be seen in the XRD patterns. Since such extra phases were not observed, one of the explanations of this could be a partial replacement of Li sites when doping by divalent cations. Li has a relatively bulky interstitial crystallographic position in NASICON capable of storing alkaliearth ions.^34^ If doping cation is going to a Li site, it should not result in a significant deformation of the structure.

### Electrochemical characterization

**Fig. 2:**
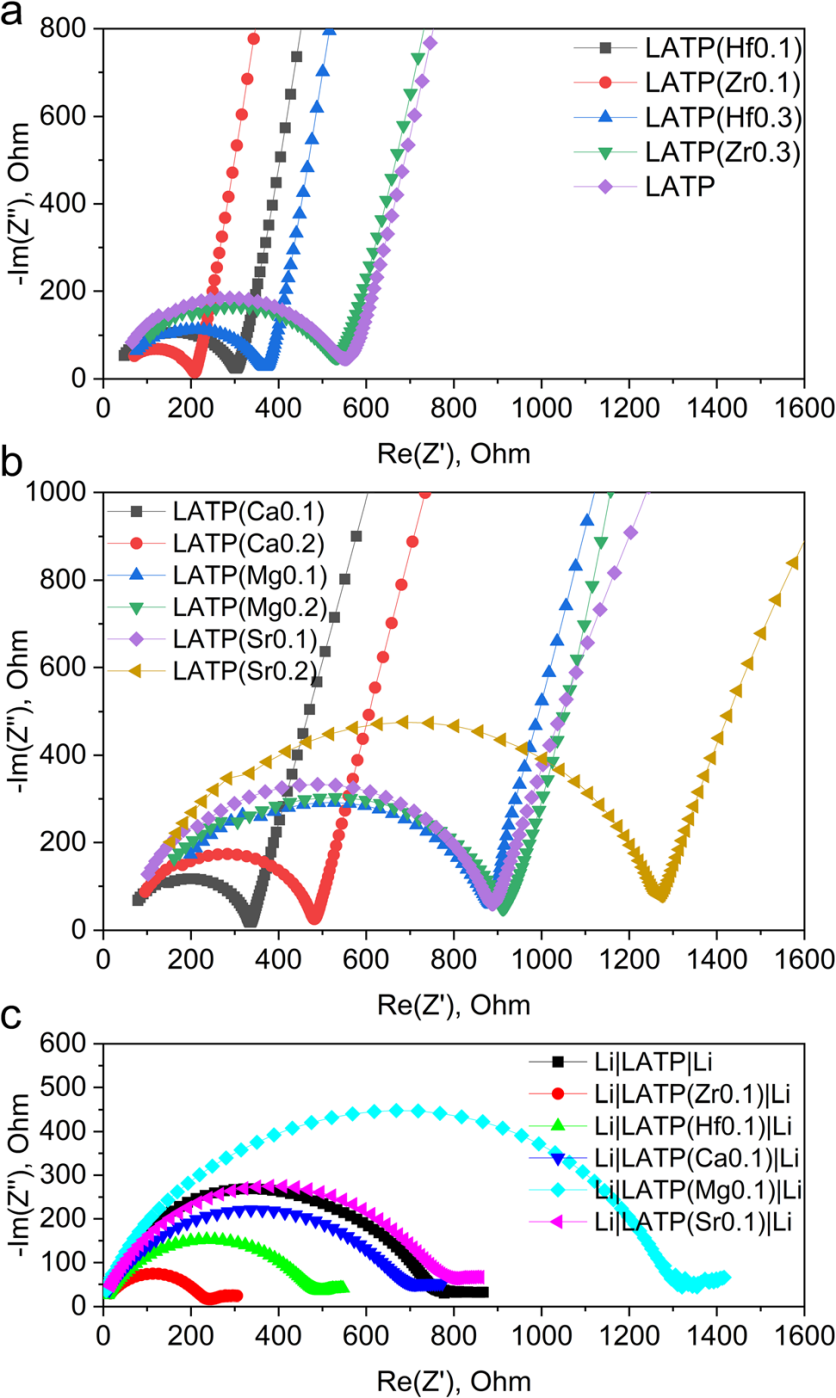
EIS spectra of LATP sample with different doping (data normalized to a pellet with an area of 1 cm^2^ and a thickness of 0.1 cm): (a) Zr, Hf doping, Au electrode cell; (b) Ca, Mg, Sr doping, Au electrode cell; (c) Zr, Hf, Ca, Sr doping, Li electrode cell.

EIS analysis was performed to investigate the ionic conductivity of doped samples, the results of which are displayed in Fig. 2a and b (fitted data Fig. S6†). The activation energy of ion migration was calculated from the EIS data retrieved in the temperature range of 25–100 °C. The calculated values of ionic conductivity and activation energy of ion migration are summarized in Table 2.

**Table 2:** The calculated ionic conductivity and density of the samples

Sample	Ionic conductivity at 40 °C, S cm^−1^	Density, % of the theoretical	Activation energy of ion migration, eV	Ionic conductivity after contact with Li at 40 °C, S cm^−1^
LATP	1.80 × 10^−4^	91	0.45	7.86 × 10^−5^
LATP(Zr_0.1_)	4.07 × 10^−4^	91	0.47	4.70 × 10^−5^
LATP(Zr_0.3_)	1.84 × 10^−4^	90	0.53	—
LATP(Hf_0.1_)	2.68 × 10^−4^	94	0.46	2.41 × 10^−5^
LATP(Hf_0.3_)	2.69 × 10^−4^	93	0.51	—
LATP(Mg_0.1_)	1.13 × 10^−4^	90	0.74	3.01 × 10^−6^
LATP(Mg_0.2_)	1.00 × 10^−4^	89	0.82	—
LATP(Ca_0.1_)	2.80 × 10^−4^	93	0.48	9.02 × 10^−5^
LATP(Ca_0.2_)	2.10 × 10^−4^	89	0.61	—
LATP(Sr_0.1_)	1.10 × 10^−4^	92	0.46	6.91 × 10^−5^
LATP(Sr_0.2_)	8.10 × 10^−5^	88	0.62	—

Since the conductivity measurements are affected by a large number of variables, an initial undoped LATP again acted as a reference point. The undoped sample demonstrated 1.8 × 10^−4^ S cm^−1^ with the pellet density 91% of the theoretical being an order of magnitude lower than expected from the literature.^13^ From Table 2, interestingly the doping with both types of cations do not change the conductivity of the material significantly suggesting that the ion transportation pathways in the crystal lattice are unaffected. A more noticeable and expected correlation is observed with the density of the pellet: samples with higher density exhibit a higher conductivity. However, even considering this observation it is difficult to speculate on the significant differences between samples due to the limitations of EIS measurements, where many variables are affecting the total conductivity. The conductivity variation range is nearly only 3 units wide (0.8–4.1 × 10^−4^ S cm^−1^). Therefore, it seems that the partial replacement of the cations did not change the conductivity of the system.

As it can be noted from Table 2, the activation energy of pristine LATP is around 0.45 eV, which is slightly higher than the value calculated by Liu J. *et al.* for an ideal crystal (0.37 eV).^35^ Additionally, the results clearly demonstrate that any replacement of the cations leads to the increase of the activation energy. The demonstrated differences can be related to the changes of the energy landscape in the unit cell, or other internal factors that can facilitate the movement of the Li-ions in the doped LATPs affecting ionic mobility compared to the reference sample.^35^

### Characterization after Li contact

For all-solid-state batteries, the interfaces well-matched both physically and chemically play a key role for reaction kinetics. As it is mentioned above, the presence of Ti^4+^ causes significant obstacles for future commercial use of LATP-based electrolytes due to its reduction upon the interaction with Li and formation of an ionically isolative layer between the electrolyte and the electrode. Since Li metal is considered as the most desirable anode, Ti^4+^ in LATP is supposed to be affected by extensive reduction; therefore, the modified LATP materials should be thoroughly checked in terms of this issue.

In order to verify if the Ti^4+^ reduction rate is changed for the doped systems, the symmetrical cells were constructed with the LATP pellets sandwiched between two Li metal electrodes. According to Table 2, there is no positive dependency of the ionic conductivity on the LATP's doping level. Therefore, the samples with the lowest doping levels were taken for the symmetrical cell tests. After 20 h of dense Li contact, the EIS spectra of the cells were measured (Fig. 2c). According to the obtained Nyquist plots, the resistance increased in all cases, suggesting that the interaction between Li metal and the electrolyte formed a layer of less conductive decomposition product. Interestingly, the conductivity degradation was rather similar for all the samples suggesting the same mechanism.

**Fig. 3:**
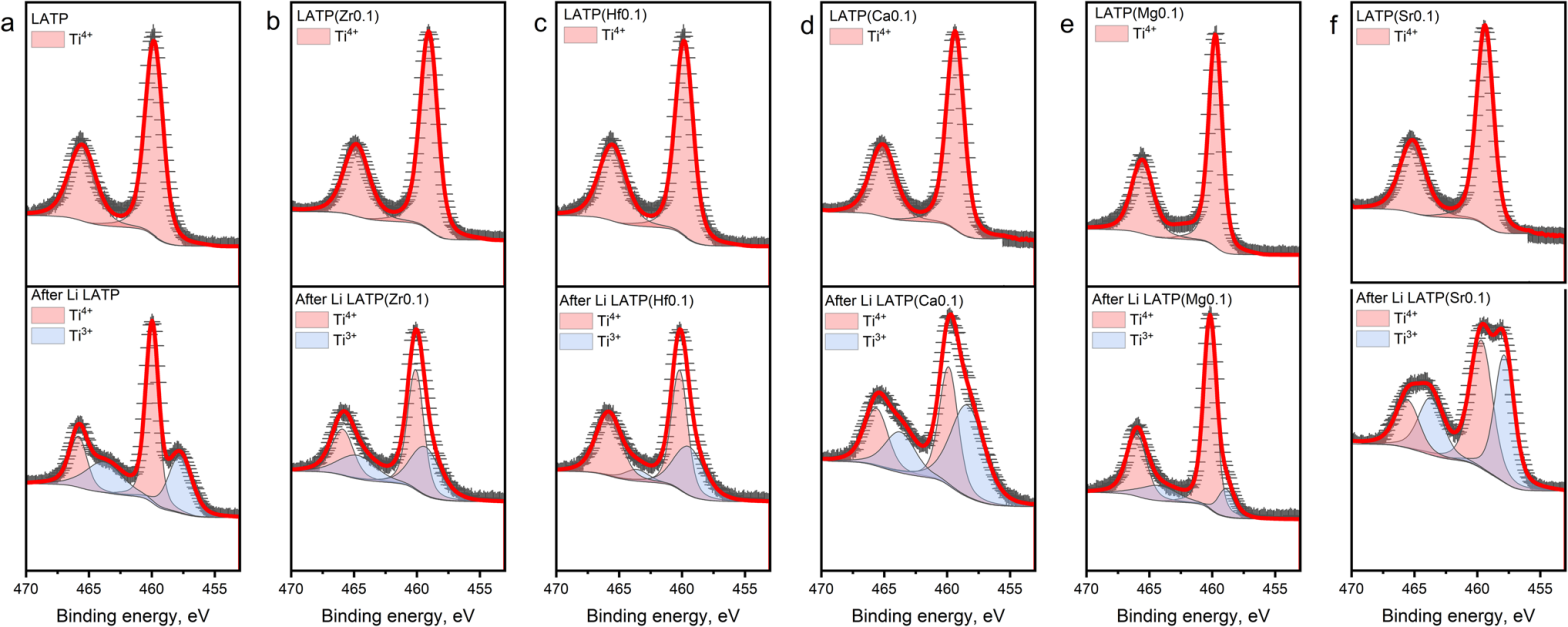
XPS spectra of Ti 2p before and after contact with Li. The XPS spectra of fresh samples located at the top, the spectra of the samples after the contact for 20 h are at the bottom. Red line—calculated profile, black crosses—experimental data, red area—Ti^4+^, blue area—Ti^3+^. (a) LATP; (b) LATP(Zr_0.1_); (c) LATP(Hf_0.1_); (d) LATP(Ca_0.1_), (e) LATP(Mg_0.1_) and (f) LATP(Sr_0.1_).

The oxidation states of Ti in the structure before and after the contact with Li metal were analysed using XPS (Fig. 3). The pristine LATP spectra (Fig. 3a) show two dominant peaks located at ∼458.7 and ∼464.4 eV corresponding to Ti 2p_1/2_ and Ti 2p_3/2_, respectively, indicating the presence of Ti^4+^.^37^ The Ti 2p_1/2_ and Ti 2p_3/2_ peaks of all fresh doped electrolytes, which illustrated in Fig. 3b–f, shifted by ∼0.8 and ∼1.1 eV, respectively, towards higher binding energies (Table S1†). Such a difference can be related to a partial substitution of Ti, which may decrease the number of outlet electrons around an atom forming a stronger electron delocalization.^37^ The *ex situ* XPS spectra of the samples after the contact with Li metal demonstrate two additional peaks at the smaller binding energies which corresponds to the presence of Ti^3+^.^37–39^ As described above, two different methods were used for cation doping synthesis, but it should be noted that no nano-structural frameworks were formed, so that Ti reduction is not promoted by a synthesis route but rather by the presence of substituting cations in the structure. It can be seen from the bottom part of Fig. 3b and c that the peaks corresponding to Ti^3+^ have a smaller corresponding area than that for pristine LATP, which may indicate a slightly lower reduction of Ti in the LATP(Zr_0.1_) and LATP(Hf_0.1_) samples after the contact with Li. From Fig. 3d–f, the XPS spectra of LATP(Ca_0.1_) and LATP(Sr_0.1_) samples, it can clearly be seen that the peaks corresponding to Ti^3+^ are much larger than those of pure LATP (Table S1†). The XPS observation revealed that the interaction with Li metal led to the Ti^4+^ reduction of a greater extent in case of the Ca^2+^ and Sr^2+^ dopants. The lower reduction tolerance might appear from a larger ionic size mismatch between Ca^2+^ (*r* = 1 Å), Sr^2+^ (*r* = 1.18 Å) and Ti^4+^ (*r* = 0.6 Å).

**Fig. 4:**
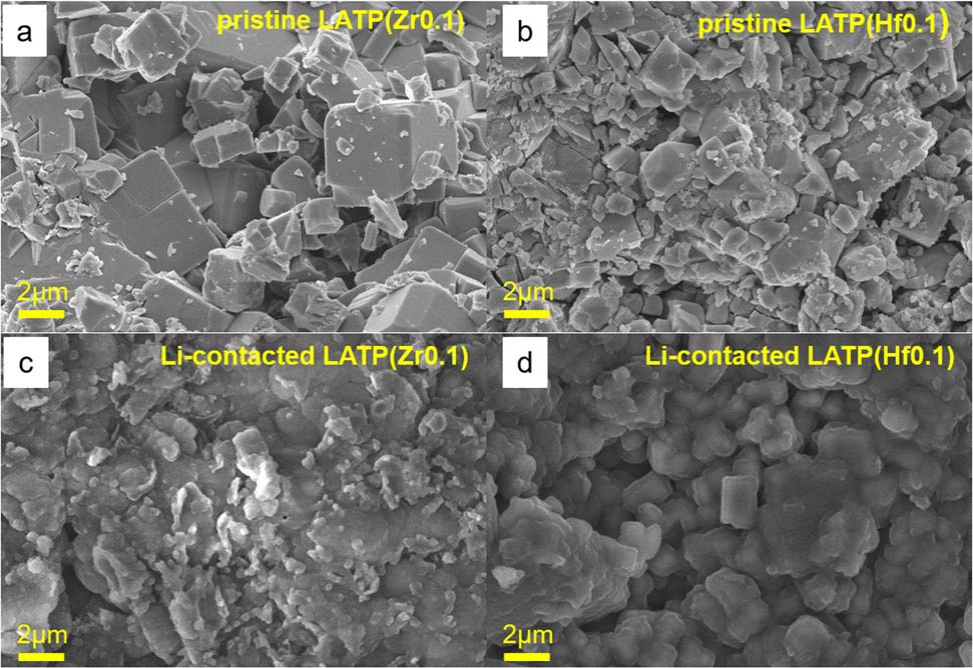
SEM images of doped electrolytes before and after contact with lithium metal: (a) LATP(Zr_0.1_), (b) LATP(Hf_0.1_), (c) after Li contact, LATP(Zr_0.1_)/Li, (d) after Li contact, LATP(Hf_0.1_)/Li.

To obtain the information on the effect of Li contact on the morphology degradation, the cells were disassembled after the EIS measurements. The morphology of the electrolytes was studied and compared with the initial electrolyte morphology (Fig. 4 and S5†). Fig. 4a and b demonstrate the initial morphology of the Zr and Hf doped solid electrolytes with similar square shaped particles with distinct facets. The average size of the LATP(Zr) and LATP(Hf) electrolyte particles is about 1.5 μm with some agglomerates of pronounced large (2 μm) crystals. Presumably, the formation of such agglomerates is associated with the method of the electrolyte synthesis. Fig. 4c and d show that after the contact with Li the electrolytes particles are still homogeneously distributed. However, the crystallinity of the particles decreased and the crystal blocks are not defined as well as in the fresh samples (Fig. 4a and b).

Interestingly, the initial higher crystallinity of the samples is more related to the comparable ionic sizes of the doping cations to Ti^4+^. Zr^4+^, Hf^4+^, and Mg^2+^ having closer ionic radii produce more clearly defined crystal blocks, while the Ca and Sr doped samples demonstrate more amorphous morphology (Fig. S5†). Apparently, much larger Ca^2+^ and Sr^2+^ cations introduce a significant amount of microstrain in the crystal lattice, therefore causing various defects at the macro level while leaving local crystallinity the same, thus non-prominent on the XRD patterns.^36^

The defects caused by this mismatch result in the less crystalline surface and likely in a lower potential barrier for Ti^4+^ reduction. More size-similar ions like Mg^2+^, Zr^4+^, Hf^4+^ do not have such an effect on the Ti^4+^ reduction tolerance and, therefore, can be further studied.

## Conclusions

The NASICON-type LATP ceramic solid electrolyte was doped by heterovalent dopants with preserving the Al content using the molten flux and solid-state method. The influence of different dopants and their content on the ionic conductivity and Ti^4+^ reduction of the modified LATP solid electrolytes was systematically studied. We demonstrated that a small ratio doping does not significantly affect the conductivity while modifies its redox abilities. The tetravalent cations with similar sizes (Zr^4+^, Hf^4+^) seem to be suppressing the Ti^4+^ reduction while the bulky divalent cations (Ca^2+^, Sr^2+^) promote it even at relatively low concentrations. Mg^2+^ while showing improvement in the Ti^4+^ reduction tolerance demonstrated a detrimental effect on the conductivity after the contact with Li metal. This phenomenon unravels a vulnerability of LATP to divalent cation impurities. The importance of this discovery urges the raising of the requirements for potential industrial LATP production, where divalent cations impurities should be carefully eliminated during the synthesis stage in order to achieve the better-quality product. Additionally, we found that the role of morphology and crystallinity of the LATP surface might play a critical role not only as an electrode–electrolyte diffusion system but also as a variable for potential catalytic properties.

The reported work provides the important synthesis details and scientific findings to design the LATP-based solid electrolytes that are high-ionically conductive and less sensitive to Ti^4+^ reduction.

## Author contributions

Aiym Mashekova: conceptualization; experiments; methodology, overall; investigation; writing—original draft preparation; visualization. Yelnury Baltash: experiments; writing—Sections 2 and 3; writing, revision and editing. Mukagali Yegamkulov: experiments. Ivan Trussov: conceptualization; methodology, overall; resources; validation; investigation; data curation; writing-revision and editing. Zhumabay Bakenov: analysis, resources, proofreading and editing. Aliya Mukanova: conceptualization; supervision; resources; funding acquisition; analysis; writing-review and editing.

## Conflicts of interest

The authors declare no conflicts of interest.

## Supplementary Material

RA-012-D2RA05782D-s001
