# Supplementary material for: Polycationic doping of the LATP ceramic electrolyte for Li-ion batteries
Source: RSC Adv. 2022 Oct 17;12(46):29595–601. doi: 10.1039/d2ra05782d (PMC9574870; doi:10.1039/d2ra05782d)
Supplement: RA-012-D2RA05782D-s001 [file RA-012-D2RA05782D-s001.pdf]

Electronic Supplementary Material (ESI) for RSC Advances.
This journal is © The Royal Society of Chemistry 2022

Please do not adjust margins

**Supplementary information**

Polycationic doping of the LATP ceramic electrolyte for lithium-ion batteries

Aiym Mashekova[a,b], Yelnury Baltash[a], Mukagali Yegamkulov[a,b], Ivan Trussov[a], Zhumabay Bakenov [a,b,c]*, Aliya Mukanova[a,b,c,] *

[a.] *Institute of Batteries LLC, Kabanbay Batyr Ave. 53, Nur-Sultan 010000, Kazakhstan;*
[b.] *Department of Chemical and Materials Engineering, School of Engineering and Digital Sciences, Nazarbayev University, Kabanbay Batyr Ave. 53, Nur-Sultan 010000, Kazakhstan;*
[c.] *National Laboratory Astana, Nazarbayev University, Kabanbay Batyr Ave. 53, Nur-Sultan 010000, Kazakhstan;*

†*Electronic Supplementary Information (ESI) available. See DOI: 10.1039/x0xx00000x*

Correspondence: aliya.mukanova@nu.edu.kz, zbakenov@nu.edu.kz

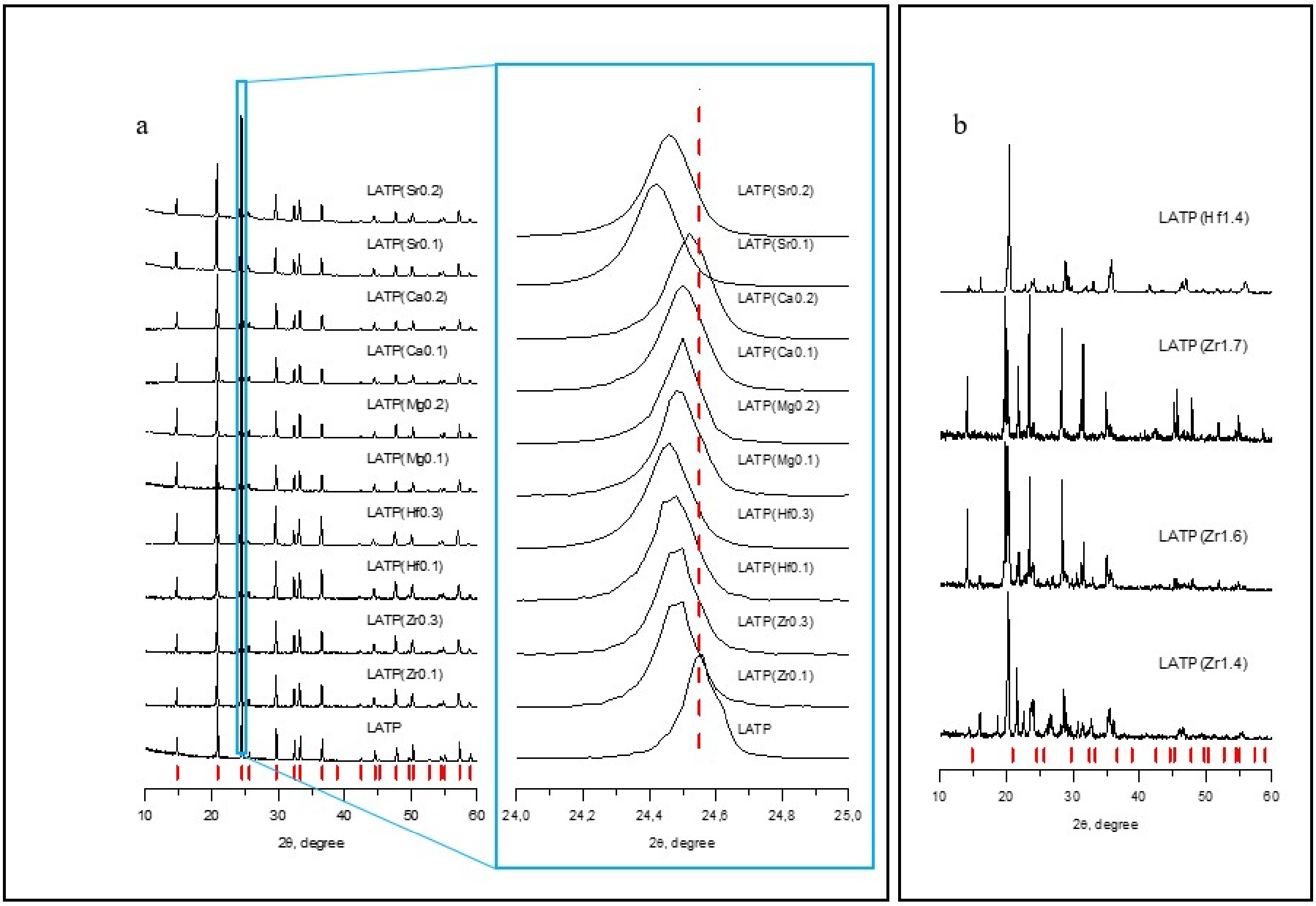


**Figure S1.** Diffraction patterns of doped LATP samples with various levels of cation substitutions: (a) XRD patterns with enlarged peaks around 2θ≈24,5$^{o}$, (b) XRD patterns with higher tetravalent cation substitution. Red tick marks represent NASICON phase with space group R-3c.

Please do not adjust margins

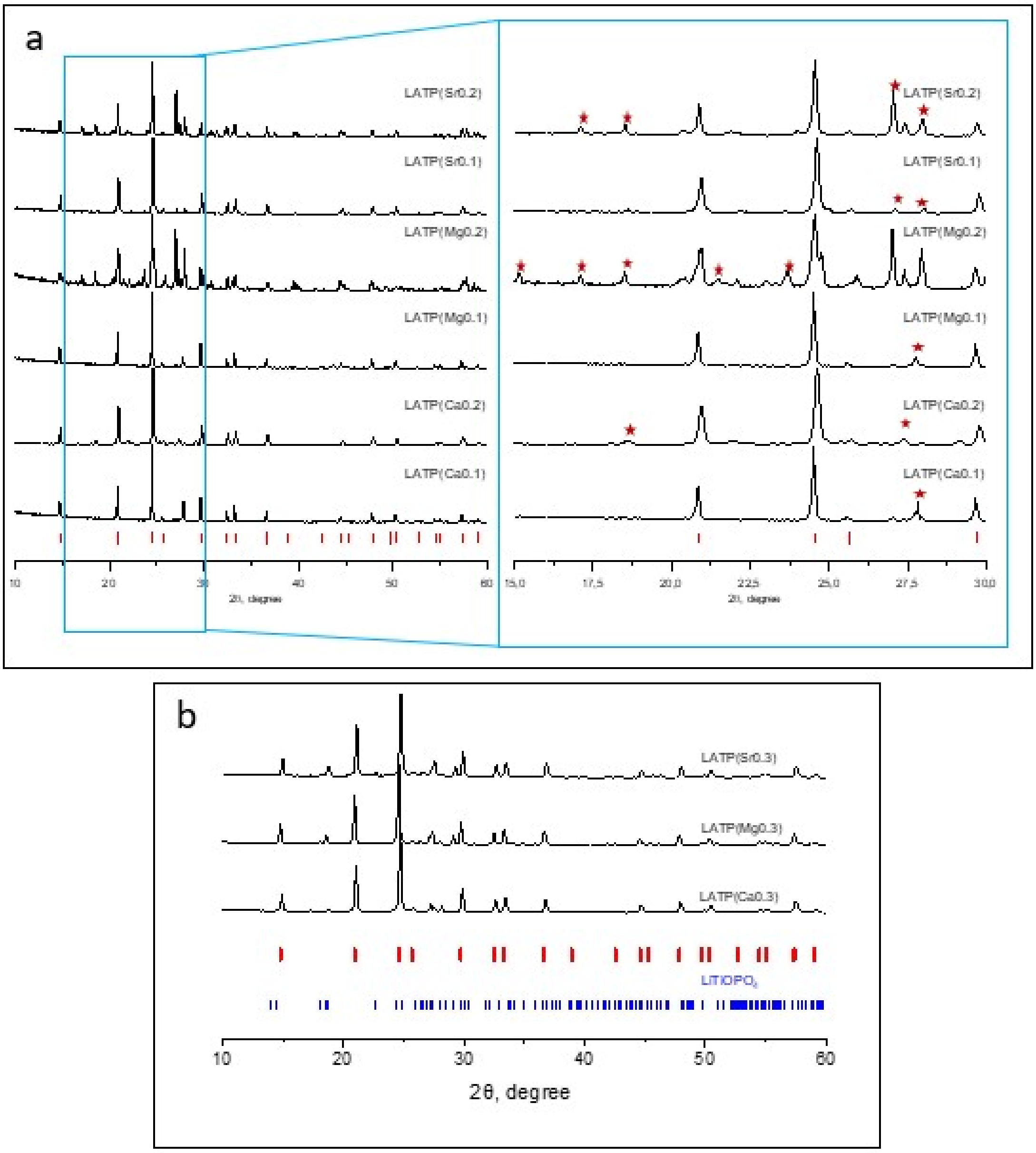


**Figure S2.** Diffraction patterns of doped LATP samples with divalent cations: (a)synthesized by the molten flux method and (b) doping cation is above 0.2 units per formula with solid-state method. The red ticks indicate the NASICON phase with the spatial group R-3c, the red asterisks the impurity phase belonging to $TiP_2O_7$

 This journal is © The Royal Society of Chemistry 20xx

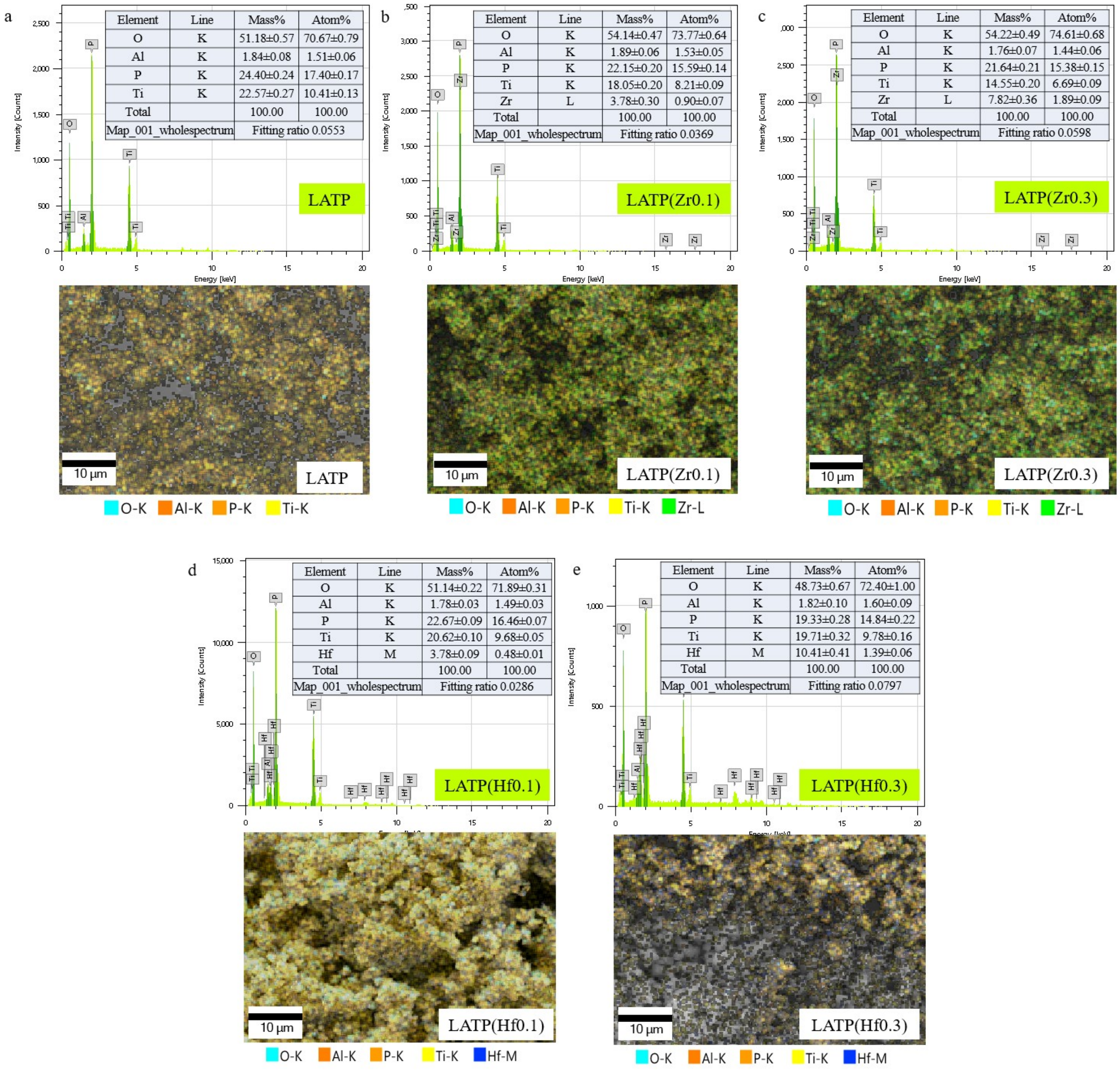


**Figure S3.** EDS spectra of electrolytes: (a) LATP, (b) LATP(Zr0.1), (c) LATP(Zr0.3), (d) LATP(Hf0.1), (e) LATP(Hf0.3)

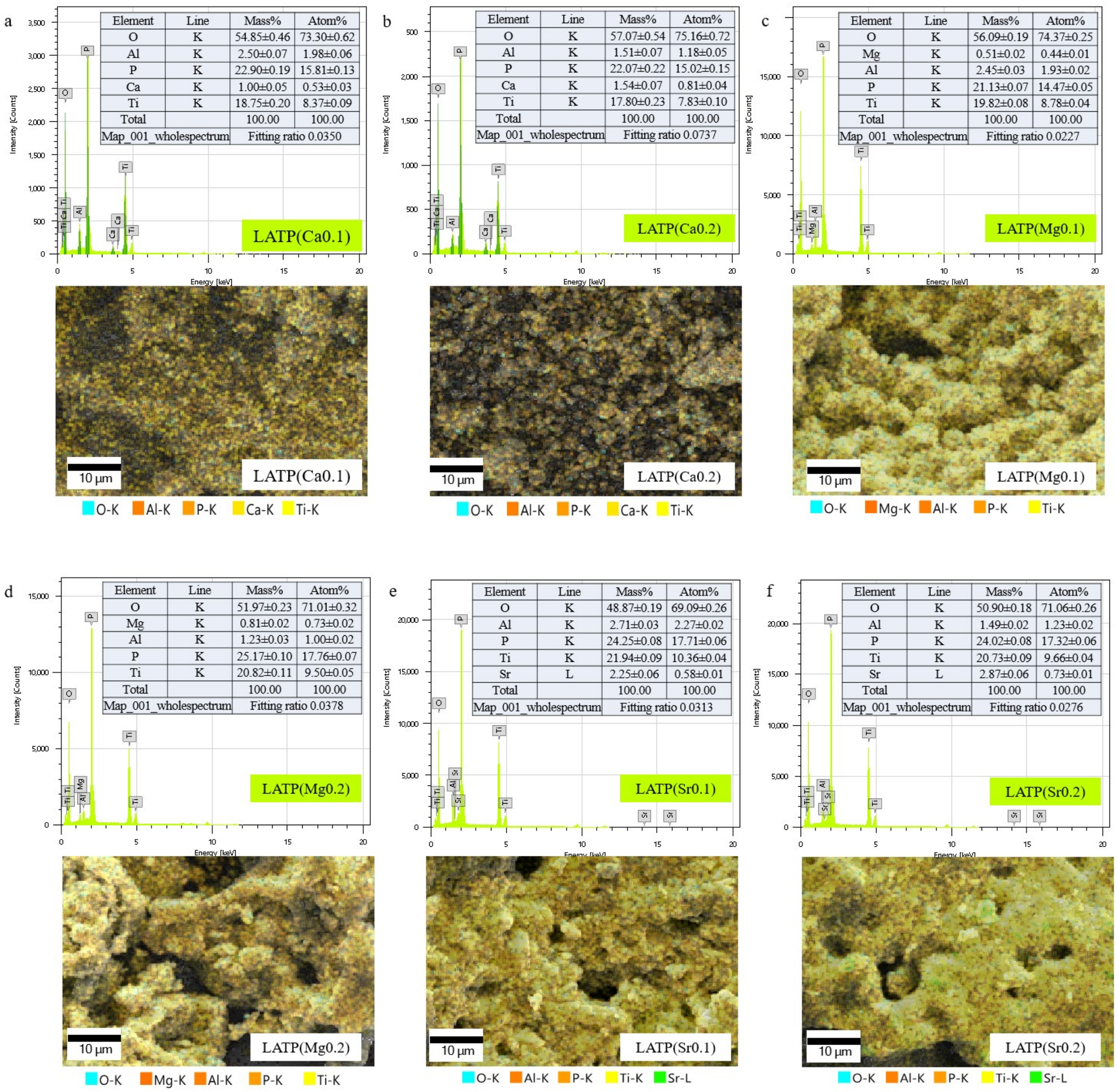


**Figure S4.** EDS spectra of electrolytes: (a) LATP(Ca0.1), (b) LATP(Ca0.2), (c) LATP(Mg0.1), (d) LATP(Mg0.2), (e) LATP(Sr0.1), (f) LATP(Sr0.2)



This journal is © The Royal Society of Chemistry 20xx

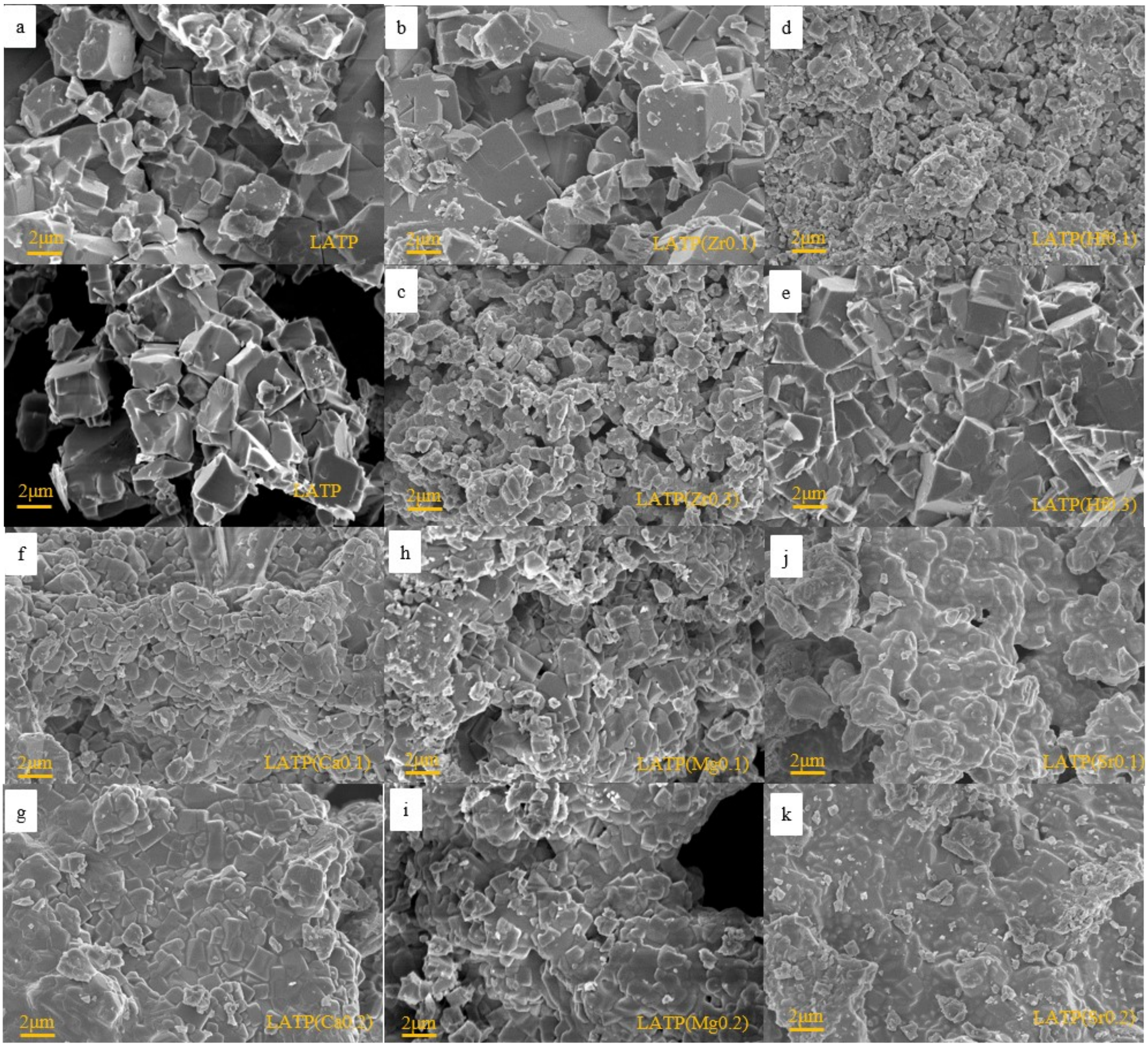


**Figure S5.** SEM images of doped LATP electrolytes: (a) LATP, (b) LATP(Zr0.1), (c) LATP(Zr0.3), (d) LATP(Hf0.1), (e) LATP(Hf0.3), (f) LATP(Ca0.1), (g) LATP(Ca0.2), (h) LATP(Mg0.1), (i) LATP(Mg0.2), (j) LATP(Sr0.1), (k) LATP(Sr0.2)

Table S1 Experimental data of XPS peaks

| Sample | Ion | Peak BE | Height CPS | FWHM eV | Area | Ti ratio, % | Sample | Ionic conductivity at 25 °C, $S\cdot cm^{-1}$ | Ion | Peak BE | Height CPS | FWHM eV | Area | Ti ratio, % |
|---|---|---|---|---|---|---|---|---|---|---|---|---|---|---|
| LATP | $Ti^{4+}_{1/2}$ | 459,83 | 21212 | 1,71 | 42558 | 100% | LATP-Li | $7.86\cdot10^{-5}$ | $Ti^{4+}_{1/2}$ | 459,98 | 45742 | 1,22 | 65552 | 56% |
| | $Ti^{4+}_{3/2}$ | 465,53 | 8357 | 2,55 | 24978 | | | | $Ti^{4+}_{3/2}$ | 465,83 | 13545 | 1,61 | 25604 | |
| | $Ti^{3+}_{1/2}$ | 0 | 0 | 0 | 0 | 0% | | | $Ti^{3+}_{1/2}$ | 457,73 | 15215 | 2,23 | 39814 | 44% |
| | $Ti^{3+}_{3/2}$ | 0 | 0 | 0 | 0 | | | | $Ti^{3+}_{3/2}$ | 463,79 | 8099 | 3,5 | 33256 | |
| LATP(Zr0.1) | $Ti^{4+}_{1/2}$ | 459,05 | 62802 | 1,67 | 122900 | 100% | LATP(Zr0.1)-Li | $4.7\cdot10^{-5}$ | $Ti^{4+}_{1/2}$ | 460,1 | 11643 | 1,53 | 21899 | 60% |
| | $Ti^{4+}_{3/2}$ | 464,8 | 22937 | 2,39 | 64297 | | | | $Ti^{4+}_{3/2}$ | 465,91 | 4488 | 2,05 | 9774 | |
| | $Ti^{3+}_{1/2}$ | 0 | 0 | 0 | 0 | 0% | | | $Ti^{3+}_{1/2}$ | 459,33 | 4725 | 2,73 | 14128 | 40% |
| | $Ti^{3+}_{3/2}$ | 0 | 0 | 0 | 0 | | | | $Ti^{3+}_{3/2}$ | 464,73 | 2292 | 3,5 | 7012 | |
| LATP(Hf0.1) | $Ti^{4+}_{1/2}$ | 459,78 | 11171 | 1,55 | 37562 | 100% | LATP(Hf0.1)-Li | $2.41\cdot10^{-5}$ | $Ti^{4+}_{1/2}$ | 460,19 | 11607 | 1,53 | 20834 | 67% |
| | $Ti^{4+}_{3/2}$ | 465,58 | 4595 | 2,26 | 22505 | | | | $Ti^{4+}_{3/2}$ | 465,84 | 6061 | 2,38 | 16910 | |
| | $Ti^{3+}_{1/2}$ | 0 | 0 | 0 | 0 | 0% | | | $Ti^{3+}_{1/2}$ | 459,44 | 4734 | 2,78 | 15458 | 33% |
| | $Ti^{3+}_{3/2}$ | 0 | 0 | 0 | 0 | | | | $Ti^{3+}_{3/2}$ | 463,59 | 1120 | 2,27 | 2976 | |
| LATP(Ca0.1) | $Ti^{4+}_{1/2}$ | 459,35 | 62772 | 1,66 | 122074 | 100% | LATP(Ca0.1)-Li | $9.02\cdot10^{-5}$ | $Ti^{4+}_{1/2}$ | 459,87 | 8762 | 1,77 | 18205 | 45% |
| | $Ti^{4+}_{3/2}$ | 465,11 | 23017 | 2,42 | 65312 | | | | $Ti^{4+}_{3/2}$ | 465,62 | 4277 | 2,19 | 10998 | |
| | $Ti^{3+}_{1/2}$ | 0 | 0 | 0 | 0 | 0% | | | $Ti^{3+}_{1/2}$ | 458,38 | 6807 | 3,11 | 24866 | 55% |
| | $Ti^{3+}_{3/2}$ | 0 | 0 | 0 | 0 | | | | $Ti^{3+}_{3/2}$ | 463,74 | 3017 | 2,89 | 10238 | |
| LATP(Mg0.1) | $Ti^{4+}_{1/2}$ | 459,74 | 73804 | 1,26 | 109327 | 100% | LATP(Mg0.1)-Li | $3,01\cdot10^{-6}$ | $Ti^{4+}_{1/2}$ | 460,18 | 43139 | 1,22 | 61842 | 78% |
| | $Ti^{4+}_{3/2}$ | 465,56 | 25646 | 2,03 | 61000 | | | | $Ti^{4+}_{3/2}$ | 465,98 | 14095 | 1,86 | 30786 | |
| | $Ti^{3+}_{1/2}$ | 0 | 0 | 0 | 0 | 0% | | | $Ti^{3+}_{1/2}$ | 458,93 | 6441 | 1,49 | 11282 | 22% |
| | $Ti^{3+}_{3/2}$ | 0 | 0 | 0 | 0 | | | | $Ti^{3+}_{3/2}$ | 464,27 | 3484 | 3,5 | 14306 | |
| LATP(Sr0.1) | $Ti^{4+}_{1/2}$ | 459,38 | 64171 | 1,62 | 122232 | 100% | LATP(Sr0.1)-Li | $6.91\cdot10^{-5}$ | $Ti^{4+}_{1/2}$ | 459,67 | 25205 | 2,09 | 61845 | 51% |
| | $Ti^{4+}_{3/2}$ | 465,14 | 23548 | 2,4 | 66276 | | | | $Ti^{4+}_{3/2}$ | 465,54 | 9442 | 2,19 | 24300 | |
| | $Ti^{3+}_{1/2}$ | 0 | 0 | 0 | 0 | 0% | | | $Ti^{3+}_{1/2}$ | 457,86 | 25057 | 1,82 | 53330 | 49% |
| | $Ti^{3+}_{3/2}$ | 0 | 0 | 0 | 0 | | | | $Ti^{3+}_{3/2}$ | 463,7 | 11262 | 2,34 | 30909 | |


This journal is © The Royal Society of Chemistry 20xx

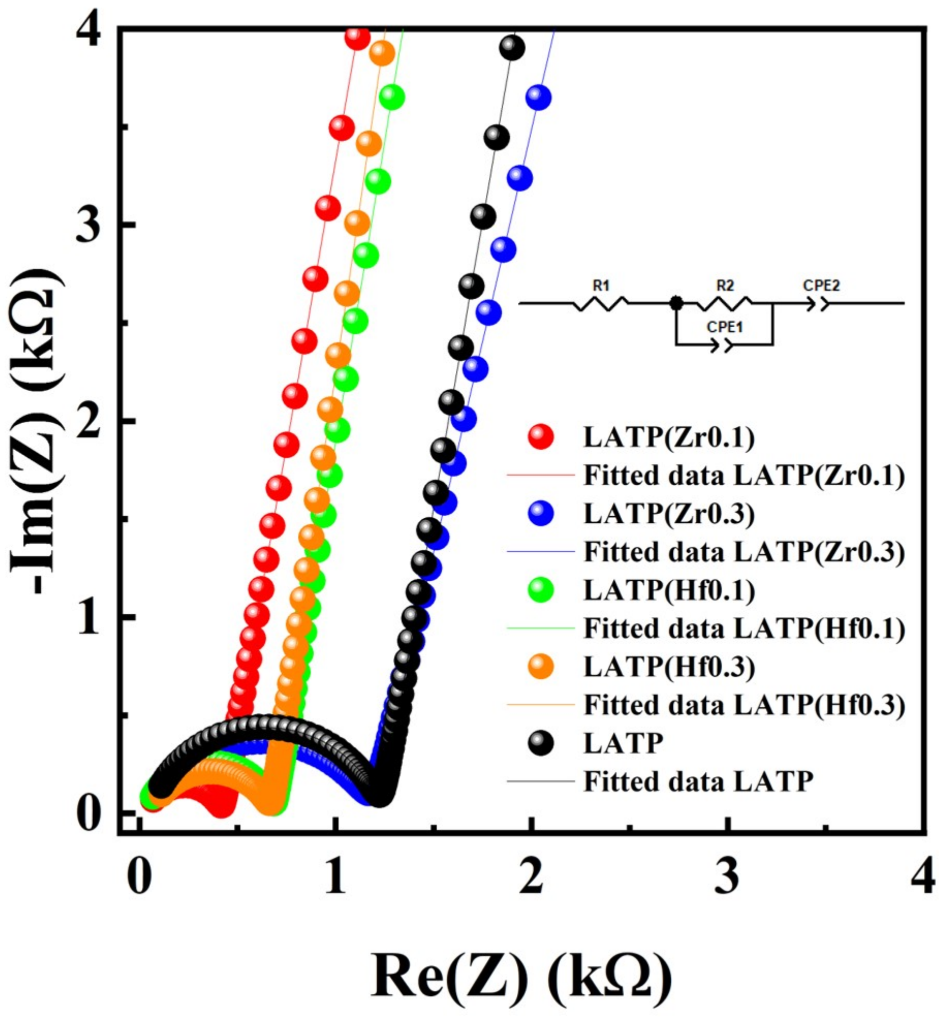


**Figure S6.** Fitted EIS results of pure and doped LATP electrolytes
